# Missing Disclaimer and Additional Information

**DOI:** 10.1001/jamanetworkopen.2026.18357

**Published:** 2026-05-29

In the Original Investigation titled “Physician Intent to Reduce Hours and Intent to Leave Organizations,”^1^ published May 6, 2026, a Disclaimer and Additional Information were missing from the Article Information. The article has been revised to indicate that the opinions offered in this article are those of the authors and should not be interpreted as American Medical Association policy (disclaimer) and that Dr Sinsky was formerly affiliated with the American Medical Association (additional information). This article has been corrected.^1^
